# Association of TRPV1 genetic variants with cognitive functions in Parkinson disease

**DOI:** 10.1016/j.isci.2025.114538

**Published:** 2025-12-24

**Authors:** Wei-Shan Yao, Rwei-Ling Yu, Chun-Hsiang Tan

**Affiliations:** 1 School of Medicine, College of Medicine, Kaohsiung Medical University, Kaohsiung, Taiwan; 2 Institute of Behavioral Medicine, College of Medicine, National Cheng Kung University, Tainan, Taiwan; 3 Department of Neurology, Kaohsiung Medical University Hospital, Kaohsiung Medical University, Kaohsiung, Taiwan; 4 Graduate Institute of Clinical Medicine, College of Medicine, Kaohsiung Medical University, Kaohsiung, Taiwan

**Keywords:** Health sciences, Medicine, Clinical genetics, Neurology, Human genetics

## Abstract

Parkinson disease (PD) is a neurodegenerative disorder characterized by motor and non-motor symptoms, including cognitive impairment. The transient receptor potential vanilloid 1 (TRPV1) channel has been implicated in neurodegenerative processes, but its contribution to cognitive variation in PD remains unclear. This study aimed to determine whether TRPV1 genetic variants influence cognitive performance and whether these effects are moderated by PD status. Three TRPV1 polymorphisms (rs8065080, rs12936340, rs182637) were genotyped in 274 healthy control subjects and 127 individuals with PD. Cognitive function was assessed across global, executive, visuospatial, memory, attention, and language domains. Moderation analysis revealed that rs12936340 and rs182637 were associated with cognitive performance, and PD status moderated these associations in specific domains. These findings suggest that TRPV1 variation relates to cognitive outcomes in PD and may contribute to individual differences in vulnerability to PD-related cognitive impairment.

## Highlights

• 401 participants (274 control subjects, 127 PD) underwent TRPV1 genotyping and cognition tests
• TRPV1 rs12936340 is associated with visuospatial function and MoCA naming
• TRPV1 rs182637 is associated with MoCA total score and MoCA attention
• PD diagnosis moderated rs12936340-naming and rs182637-attention effects

## Graphical abstract

**Figure:**
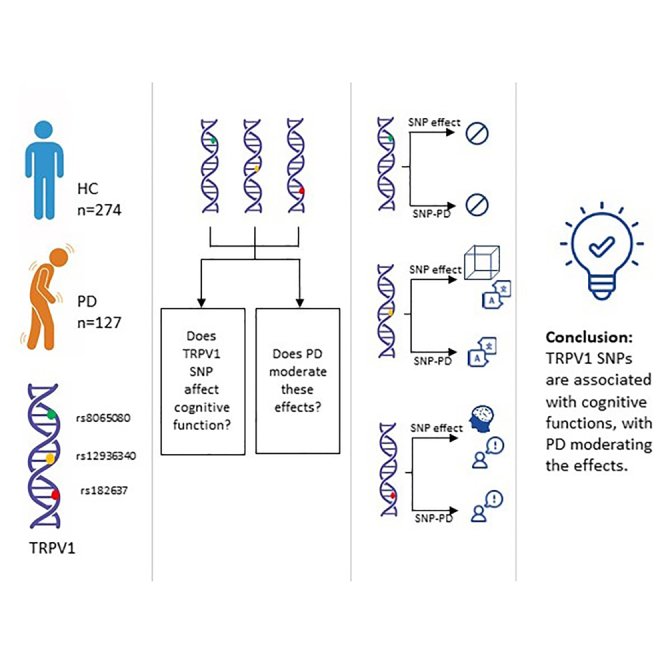

## Introduction

Parkinson disease (PD) is a progressive neurodegenerative disease with increasing prevalence in aging populations. Although motor symptoms due to dopamine depletion in the basal ganglia are the hallmark of PD, non-motor features, such as psychiatric symptoms and cognitive impairment, are equally prevalent and significantly impact patients’ quality of life.^1^^,^^2^ Cognitive deficits in PD encompass various domains, including decision-making, cognitive flexibility, reinforcement learning, and behavioral inhibition.^1^^,^^3^ Affected cognitive domains include attention, frontal-executive functions, memory, visuospatial skills, and language,^4^ further compounding the disease burden on patients, caregivers, and healthcare systems.^5^^,^^6^

The mechanisms underlying cognitive impairment in PD are not fully understood, but several pathways have been identified. The Braak hypothesis posits that the pathological protein α-synuclein propagates from brainstem neurons to cortical regions, disrupting higher-order cognitive processes.^7^ Moreover, genetic factors such as apolipoprotein E,^8^^,^^9^ microtubule-associated protein tau,^10^ glucocerebrosidase,^2^ and α-synuclein^11^ have been implicated in cognitive decline in PD. Despite these advancements, our understanding remains incomplete, underscoring the need for further exploration of novel mechanisms.

One promising target for investigation is transient receptor potential vanilloid 1 (TRPV1), a polymodal ion channel traditionally known for its role in thermosensation and pain signaling. Emerging evidence suggests that TRPV1 plays a broader role in cognitive functions and neurodegenerative diseases.^12^^,^^13^^,^^14^^,^^15^ In a rat model, activation of astrocytic TRPV1 induced the production of endogenous ciliary neurotrophic factor, thereby protecting dopaminergic neurons and facilitating behavioral recovery, suggesting a potential neuroprotective role relevant to PD pathophysiology.^16^ TRPV1 is also expressed in the hippocampus, where it contributes to excitatory innervation and spatial memory.^17^ Additionally, evidence from mouse models demonstrates that TRPV1 modulates key neuropsychological functions, including depression,^18^ anxiety,^19^ and cognitive impairment.^15^

The role of TRPV1 in neuroinflammation,^13^ a key mechanism underlying cognitive impairment in PD,^20^ is particularly compelling. This ion channel regulates intracellular calcium homeostasis and mitochondrial function,^21^ processes integral to neuroinflammatory pathways and neurodegeneration. Furthermore, TRPV1 genetic variations, including single nucleotide polymorphisms (SNPs), have been increasingly linked to various aspects of pathophysiological processes, but their contributions to cognitive dysfunction in PD remain largely unexplored.^22^^,^^23^

Given this background, the present study aims to explore the impact of TRPV1 SNPs on cognitive function and examine whether PD moderates these effects. We hypothesize that TRPV1 genetic variants distinctly impact cognitive function, with the effects potentially being moderated by PD. To test this, a moderation analysis was employed to investigate the interactions between TRPV1 SNPs and PD in shaping cognitive performance.

## Results

### Genotype distributions

**Figure 1:**
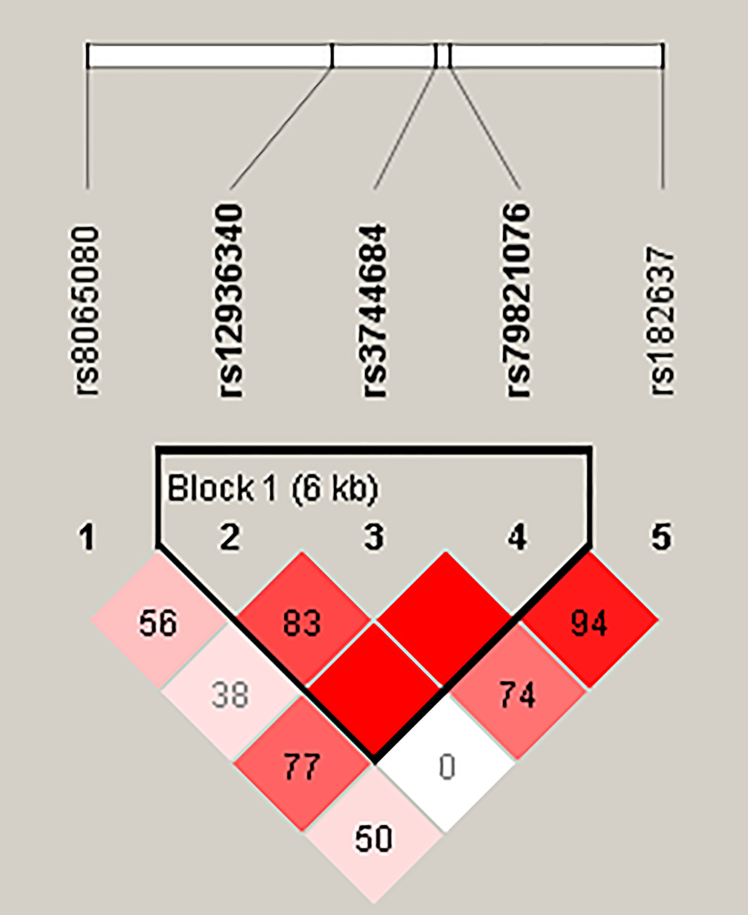
Linkage disequilibrium (LD) plot of TRPV1 single nucleotide polymorphisms (SNPs) The plot illustrates the LD between five TRPV1 SNPs: rs8065080, rs12936340, rs3744684, rs79821076, and rs182637. The strength of LD is represented by the color intensity of the diamond grid, with darker red indicating higher LD values (r^2^) and lighter shades corresponding to weaker LD. The numerical values within each diamond represent the percentage of LD (D′) between SNP pairs. Block 1, spanning a 6-kb region, highlights a region of strong LD.

A total of 27 TRPV1 SNPs were examined, and the results are shown in Table 1. Only SNPs with a minor allele frequency exceeding 10% were selected for further analysis to ensure an adequate sample size for each genotype. The genetic distributions of the selected SNPs conformed to the Hardy-Weinberg Equilibrium. Linkage disequilibrium (LD) analysis was performed for SNPs rs8065080, rs12936340, rs3744684, rs79821076, and rs182637 (Figure 1). The analysis revealed strong LD among rs12936340, rs3744684, and rs79821076, with rs12936340 exhibiting the highest minor allele frequency. Therefore, rs12936340 was selected for further analysis along with rs8065080 and rs182637. The detailed analyses of the SNPs showing strong LD with rs12936340 (rs3744684 and rs79821076) are provided in Tables S1 to S3. Of the three SNPs presented, data for the SNP rs182637 were unavailable for three participants due to no calls on the microarray.

**Table 1:** The allelic frequency of TRPV1 SNPs in all participants

SNP ID	Position	Minor/Major allele	Most severe consequence	MAF (%)	HWE (*p*-value)	Population Alt allele frequency: Alt allele	Population Alt allele frequency: Ref allele	Population Alt allele frequency: ALFA Asian	Population Alt allele frequency: ALFA total
rs16953166	3571107	C/T	Intron Variant	1.88	0.702	C	T	0.036	0.004
rs764004524	3572121	A/C	Splice Donor Variant	0	–	A	C	NDA	<0.001
rs200730890	3572123	C/T G/T	Missense Variant	0	–	C G	T	0.000 0.000	<0.001 0.000
rs200725051	3573642	T/C	Stop Gained	0	–	T	C	0.000	0.000
rs369589879	3573662	A/G	Stop Gained	0	–	A	G	NDA	<0.001
rs17706245	3573696	A/G	Synonymous Variant	3.75	0.436	A	G	0.045	0.053
rs745691159	3573957	G/T	Splice Acceptor Variant	0	–	G	T	NDA	0.000
rs8065080	3577153	T/C	Missense Variant	40.02	0.874	C	T	0.576	0.377
rs202240327	3585789	A/G	Stop Gained	0	–	A	G	0.000	0.000
rs12936340	3589704	A/G	Intron Variant	22.82	0.167	A	G	0.156	0.561
rs749802004	3589823–3589840	-/TGCCAGAGCCAGC	Frameshift Variant	0	–	–	TGCCAGAGCCAGC	0.000	<0.001
rs200444144	3590253	A/G	Synonymous Variant	0	–	A	G	0.000	<0.001
rs768635562	3590286–3590288	-/A	Frameshift Variant	0	–	delA	AAA	0.000	<0.001
rs187207058	3591328	T/C	Missense Variant	1.00	0.840	T	C	0.014	<0.001
rs751279599	3592084–3592085	GT/-	Frameshift Variant	0	–	dupGT	GT	0.000	<0.001
rs372945414	3592130	A/G	Missense Variant	0	–	A	G	0.000	<0.001
rs56095209	3592171	T/C	Synonymous Variant	2.26	0.644	T	C	0.000	0.001
rs778125374	3592286–3592288	-/G	Frameshift Variant	0	–	delG	GGG	0.000	0.000
rs3744684	3595022	G/A	Intron Variant	17.96	0.530	G	A	0.107	0.229
rs61116089	3595258	A/G	Intron Variant	2.13	0.664	A	G	0.027	0.002
rs79821076	3595800	A/G	Intron Variant	17.75	0.583	A	G	0.129	0.004
rs138089860	3598379	A/G	Intron Variant	2.00	0.683	A	G	0.036	0.002
rs75005297	3600102	A/G	Intron Variant	2.38	0.627	A	G	0.018	<0.001
rs79350035	3600261	T/C	Intron Variant	1.75	0.722	T	C	0.008	<0.001
rs61642919	3606255	T/C	Intron Variant	7.14	0.124	T	C	0.062	0.009
rs182637	3606538	T/C	Intron Variant	30.35	0.708	T	C	0.658	0.417
rs200377484	3610562	G/A	2 KB Upstream Variant	0	–	G	A	0.000	<0.001

MAF, minor allele frequency; HWE, Hardy-Weinberg Equilibrium; ALFA, allele frequency aggregator; NDA, no data available.

### Participants characteristics

We compared demographic information, including ages, sex, and years of education, of rs8065080, rs12936340, and rs182637 in the healthy control (HC) group and PD patients separately. Table 2 shows the demographic information and genotype distributions of the HC group, which included 274 participants. For SNP rs8065080, we found 172 participants in the HC group to have TT + TC genotypes and 102 participants to have CC genotypes, with no significant differences in age, sex ratio, or years of education between these groups. Regarding SNP rs12936340, we found 108 participants to have AA + AG genotypes and 166 participants to have GG genotypes, again showing no significant difference in age, sex, or years of education between these groups. Similarly, for SNP rs182637, 136 participants had CC + CT genotypes and 135 participants had TT genotypes, with no significant demographic differences between these subgroups.

**Table 2:** Effects of TRPV1 SNPs on the cognitive functions of participants in the healthy control group

SNP	rs8065080 (*n* = 274)	rs8065080 (*n* = 274)	rs8065080 (*n* = 274)	rs12936340 (*n* = 274)	rs12936340 (*n* = 274)	rs12936340 (*n* = 274)	rs182637 (*n* = 271)	rs182637 (*n* = 271)	rs182637 (*n* = 271)
Genotype	TT + TC (*n* = 172)	CC (*n* = 102)	Statistic	AA + AG (*n* = 108)	GG (*n* = 166)	Statistic	CC + CT (*n* = 136)	TT (*n* = 135)	Statistic
Age (years)	65.39 ± 6.597	64.53 ± 7.267	*p* = 0.420	65.85 ± 6.538	64.56 ± 7.023	*p* = 0.229	64.35 ± 6.798	65.69 ± 6.831	*p* = 0.122
Male/Female	49/123	25/77	*p* = 0.473	28/80	46/120	*p* = 0.745	35/101	37/98	*p* = 0.755
Education (years)	12.28 ± 3.623	12.41 ± 3.785	*p* = 0.694	11.81 ± 3.862	12.66 ± 3.524	*p* = 0.073	12.52 ± 3.682	12.12 ± 3.71	*p* = 0.286
Global cognitive screening: Montreal Cognitive Assessment	24.99 ± 3.688	25.03 ± 3.703	*p* = 0.921	24.95 ± 3.68	25.04 ± 3.702	*p* = 0.793	24.95 ± 3.922	25.07 ± 3.485	*p* = 0.917
Executive function: Color Trails Test 1	54.43 ± 24.567	55.32 ± 30.087	*p* = 0.824	55.46 ± 25.883	54.3 ± 27.294	*p* = 0.760	115.16 ± 53.084	115.91 ± 53.382	*p* = 0.770
Executive function: Color Trails Test 2	116.69 ± 50.587	113.11 ± 56.849	*p* = 0.620	122.45 ± 55.351	110.74 ± 50.933	*p* = 0.096	5.66 ± 0.647	5.63 ± 0.699	*p* = 0.517
Visuospatial function/Executive function: Alternating Trail Making/Cube/Clock (Montreal Cognitive Assessment)	4.31 ± 0.908	4.26 ± 0.922	*p* = 0.570	4.17 ± 0.932	4.38 ± 0.891	*p* = 0.032	4.26 ± 0.951	4.33 ± 0.88	*p* = 0.672
Memory: Wechsler Memory Scale-III Immediate Logical Memory	33.83 ± 11.34	33.75 ± 11.711	*p* = 0.904	33.98 ± 11.898	33.69 ± 11.197	*p* = 0.992	20.17 ± 9.449	21.4 ± 8.623	*p* = 0.630
Memory: Wechsler Memory Scale-III Delayed Logical Memory	20.65 ± 9.082	21 ± 9.163	*p* = 0.862	20.85 ± 9.183	20.73 ± 9.068	*p* = 0.950	55.95 ± 28.454	53.64 ± 25.138	*p* = 0.621
Attention: Attention (Montreal Cognitive Assessment)	5.65 ± 0.663	5.62 ± 0.704	*p* = 0.700	5.65 ± 0.631	5.63 ± 0.707	*p* = 0.878	2.38 ± 0.807	2.37 ± 0.77	*p* = 0.758
Language: Naming (Montreal Cognitive Assessment)	2.63 ± 0.692	2.73 ± 0.548	*p* = 0.479	2.7 ± 0.645	2.64 ± 0.642	*p* = 0.270	2.61 ± 0.732	2.72 ± 0.542	*p* = 0.398
Language: Sentence Repetition/Verbal Fluency (Montreal Cognitive Assessment)	2.35 ± 0.784	2.4 ± 0.787	*p* = 0.502	2.35 ± 0.801	2.38 ± 0.775	*p* = 0.829	33.18 ± 11.349	34.49 ± 11.46	*p* = 0.821

Table 3 shows the demographic information and genotype distributions for the PD group, which included 127 individuals with PD. For SNP rs8065080, we found 84 participants to have TT + TC genotypes and 43 participants to have CC genotypes, with no significant differences in age, sex, or years of education between these subgroups. For SNP rs12936340, we found 59 participants to have AA + AG genotypes and 68 participants to have GG genotypes, showing no significant difference in the demographic information between these groups. As for SNP rs182637, 70 participants had CC + CT genotypes and 57 participants had TT genotypes, with no significant differences in the demographic characteristics between these groups.

**Table 3:** Effects of TRPV1 SNPs on the cognitive functions of participants with Parkinson disease

SNP	rs8065080 (*n* = 127)	rs8065080 (*n* = 127)	rs8065080 (*n* = 127)	rs12936340 (*n* = 127)	rs12936340 (*n* = 127)	rs12936340 (*n* = 127)	rs182637 (*n* = 127)	rs182637 (*n* = 127)	rs182637 (*n* = 127)
Genotype	TT + TC (*n* = 84)	CC (*n* = 43)	Statistic	AA + AG (*n* = 59)	GG (*n* = 68)	Statistic	CC + CT (*n* = 70)	TT (*n* = 57)	Statistic
Age (years)	66.44 ± 7.341	66.23 ± 8.685	*p* = 0.887	67 ± 7.881	65.82 ± 7.723	*p* = 0.398	66.7 ± 7.908	65.96 ± 7.688	*p* = 0.599
Male/Female	56/28	29/14	*p* = 0.930	40/19	45/23	*p* = 0.847	47/23	38/19	*p* = 0.955
Education (years)	12.31 ± 3.97	12.42 ± 4.199	*p* = 0.619	13.24 ± 3.729	11.57 ± 4.151	*p* = 0.028	12.96 ± 3.965	11.6 ± 4.022	*p* = 0.067
Global cognitive screening: Montreal Cognitive Assessment	23.02 ± 3.871	23.12 ± 4.266	*p* = 0.848	23.17 ± 3.979	22.96 ± 4.031	*p* = 0.659	22.67 ± 4.152	23.53 ± 3.771	*p* = 0.275
Executive function: Color Trails Test 1	76.28 ± 48.894	67.95 ± 32.686	*p* = 0.582	76.69 ± 41.713	70.65 ± 46.231	*p* = 0.246	75.45 ± 50.063	71.01 ± 35.786	*p* = 0.95
Executive function: Color Trails Test 2	156.88 ± 109.071	138.62 ± 58.883	*p* = 0.714	148.96 ± 82.244	152.2 ± 105.792	*p* = 0.954	160.15 ± 113.609	139.09 ± 65.19	*p* = 0.362
Visuospatial function/Executive function: Alternating Trail Making/Cube/Clock (Montreal Cognitive Assessment)	3.92 ± 1.143	4.07 ± 0.961	*p* = 0.616	3.81 ± 1.137	4.1 ± 1.024	*p* = 0.131	3.9 ± 1.092	4.05 ± 1.076	*p* = 0.366
Memory: Wechsler Memory Scale-III Immediate Logical Memory	28.87 ± 11.579	29.84 ± 13.427	*p* = 0.674	30.17 ± 13.341	28.35 ± 11.13	*p* = 0.404	30.69 ± 12.132	27.37 ± 12.119	*p* = 0.128
Memory: Wechsler Memory Scale-III Delayed Logical Memory	15.92 ± 9.095	17.81 ± 10.459	*p* = 0.293	17.14 ± 10.335	16.06 ± 8.921	*p* = 0.53	17.24 ± 9.420	15.72 ± 9.790	*p* = 0.375
Attention: Attention (Montreal Cognitive Assessment)	5.4 ± 1.019	5.4 ± 0.929	*p* = 0.79	5.47 ± 0.916	5.34 ± 1.045	*p* = 0.313	5.27 ± 1.141	5.56 ± 0.732	*p* = 0.169
Language: Naming (Montreal Cognitive Assessment)	2.65 ± 0.611	2.84 ± 0.485	*p* = 0.041	2.58 ± 0.7	2.84 ± 0.409	*p* = 0.015^a^ ***d*= 0.454** **CI: -0.460∼-0.064**	2.67 ± 0.583	2.77 ± 0.567	*p* = 0.207
Language: Sentence Repetition/Verbal Fluency (Montreal Cognitive Assessment)	2.13 ± 0.847	2 ± 0.976	*p* = 0.535	2.15 ± 0.867	2.03 ± 0.914	*p* = 0.469	2 ± 0.978	2.19 ± 0.766	*p* = 0.395

a *p* < 0.0167; *d*, Cohen *d*; CI, 95% confidence interval.

### Effects of TRPV1 SNPs on the cognitive functions of participants in the healthy control group

Table 2 presents the analysis of cognitive functions in the HC group. For SNP rs8065080, there were no significant differences in global cognitive function or any of the five cognitive domains between the TT + TC and CC genotypes. Similarly, for SNP rs12936340, no significant differences were found in global cognitive function, executive function, memory, attention, or language between the AA + AG genotypes and GG genotypes. However, participants with GG genotypes showed a trend toward better performance in the visuospatial function compared with the AA + AG genotypes (*p* = 0.032). For SNP rs182637, similarly, no significant differences were observed in global cognitive function or any of the five cognitive domains between the CC + CT and TT genotypes.

### Effects of TRPV1 SNPs on the cognitive functions of participants with Parkinson disease

Table 3 presents the analysis of cognitive functions in the PD group. For SNP rs8065080, no significant differences were found between TT + TC genotypes and CC genotypes in global cognitive function, executive function, visuospatial function, memory, attention, or the Sentence Repetition and Verbal Fluency subtests of the Montreal Cognitive Assessment (MoCA). However, PD participants of SNP rs8065080 with the CC genotype demonstrated a trend of better performance in the Naming subtest of the MoCA compared with those with the TT + TC genotypes (*p* = 0.041). For SNP rs12936340, no significant differences were observed between AA + AG and GG genotypes across global cognitive function, executive function, visuospatial function, memory, or attention. Similarly, no significant differences were found in the Sentence Repetition and Verbal Fluency subtests of the MoCA between these subgroups. However, in the Naming subtest of the MoCA, PD participants of SNP rs12936340 with the GG genotypes performed significantly better than those with the AA + AG genotypes (*p* = 0.015; Cohen *d* = 0.454, 95% confidence interval [CI] = −0.460 to −0.064). For SNP rs182637, no significant differences were observed in global cognitive function or any of the five cognitive domains between the CC + CT and TT genotypes in PD patients.

### Moderation between TRPV1 SNPs and Parkinson disease entity on cognitive functions

To evaluate the impact of TRPV1 SNPs on cognitive function between the HC group and the PD patients, a moderation analysis was conducted. Table 4 presents the results of the moderation analysis, examining the interaction between TRPV1 SNPs and the presence of PD in shaping cognitive functions across various cognitive domains. Moderation analysis revealed no significant association between TRPV1 SNP rs8065080 and cognitive functions. Furthermore, the diagnosis of PD did not significantly alter the association between rs8065080 and cognitive functions across global cognitive function and various cognitive domains.

**Table 4:** Moderation between TRPV1 SNPs and disease entity on cognitive functions

	rs8065080 SNP effect	rs8065080 SNP-diagnosis interaction	rs12936340 SNP effect	rs12936340 SNP-diagnosis interaction	rs182637 SNP effect	rs182637 SNP-diagnosis interaction
Global cognitive screening: Montreal Cognitive Assessment	*p* = 0.934	*p* = 0.785 1-β = 0.020	*p* = 0.344	*p* = 0.169 1-β = 0.156	*p* = 0.015^a^ **β= 0.815** **CI: 0.158 ∼1.471**	*p* = 0.165 1-β = 0.159
Executive function: Color Trails Test 1	*p* = 0.128	*p* = 0.105 1-β = 0.222	*p* = 0.061	*p* = 0.043 1-β = 0.363	*p* = 0.227	*p* = 0.825 1-β = 0.020
Executive function: Color Trails Test 2	*p* = 0.129	*p* = 0.201 1-β = 0.133	*p* = 0.964	*p* = 0.906 1-β = 0.017	*p* = 0.021	*p* = 0.133 1-β = 0.193
Visuospatial function/Executive function: Alternating Trail Making/Cube/Clock (Montreal Cognitive Assessment)	*p* = 0.343	*p* = 0.244 1-β = 0.111	*p* = 0.003^a^ **β=0.271** **CI: 0.093∼0.449**	*p* = 0.039 1-β = 0.375	*p* = 0.071	*p* = 0.402 1-β = 0.062
Memory: Wechsler Memory Scale-III Immediate Logical Memory	*p* = 0.647	*p* = 0.546 1-β = 0.040	*p* = 0.776	*p* = 0.350 1-β = 0.075	*p* = 0.268	*p* = 0.070 1-β = 0.288
Memory: Wechsler Memory Scale-III Delayed Logical Memory	*p* = 0.241	*p* = 0.299 1-β = 0.087	*p* = 0.718	*p* = 0.354 1-β = 0.071	*p* = 0.637	*p* = 0.163 1-β = 0.163
Attention: Attention (Montreal Cognitive Assessment)	*p* = 0.930	*p* = 0.810 1-β = 0.020	*p* = 0.923	*p* = 0.505 1-β = 0.045	*p* = 0.001^a^ **β= -0.046** **CI: -0.070∼ -0.021**	*p* = 0.011^a^ 1-β = 0.572 **β= -0.038** **CI: -0.068 ∼-0.009**
Language: Naming (Montreal Cognitive Assessment)	*p* = 0.142	*p* = 0.509 1-β = 0.042	*p* < 0.001^a^ ***β*= 0.216** **CI: 0.095∼ 0.337**	*p* < 0.001^a^ 1-β = 0.905 **β= -0.047** **CI: -0.070 ∼-0.021**	*p* = 0.186	*p* = 0.847 1-β = 0.019
Language: Sentence Repetition/Verbal Fluency (Montreal Cognitive Assessment)	*p* = 0.303	*p* = 0.348 1-β = 0.075	*p* = 0.915	*p* = 0.822 1-β = 0.019	*p* = 0.0411	*p* = 0.163 1-β = 0.163

a *p* < 0.0167; *β*, standardized regression coefficient; 1 – β, statistical power (where β represents the probability of a type II error); CI, 95% confidence interval.

In contrast, several significant associations were identified for SNP rs12936340. In the Color Trails Test 1, which assesses executive function, although the SNP effect did not reach statistical significance (*p* = 0.0608), a borderline significant SNP-diagnosis interaction (*p* = 0.0425) was observed, suggesting that the association between rs12936340 and executive performance may differ between the participants with PD and those in the HC group. This effect achieved nominal significance (*p* < 0.05) but did not withstand Bonferroni correction (*p* < 0.0167) and thus should be interpreted with caution. For visuospatial function, rs12936340 demonstrated a significant main effect (*p* = 0.003; effect size β coefficient = 0.271, 95% CI = 0.093 to 0.449) and a borderline SNP-diagnosis interaction (*p* = 0.039), indicating that this SNP was significantly associated with visuospatial function, with a potential moderating influence of PD. Again, the interaction effect was nominally significant but did not survive the Bonferroni correction. In the MoCA Naming subtest, rs12936340 showed a significant main effect (*p* < 0.001; effect size β coefficient = 0.216, 95% CI = 0.095 to 0.337) and a significant SNP-diagnosis interaction (*p* < 0.001; effect size β coefficient = −0.047, 95% CI = −0.070 to −0.021), suggesting that SNP rs12936340 was associated with the naming ability and that this association was significantly moderated by PD status (Figure S1).

Several significant associations were also observed for SNP rs182637. A main effect of rs182637 on global cognitive function was identified (*p* = 0.0152; effect size β coefficient = 0.815, 95% CI = 0.158 to 1.471), although no significant interaction with PD was detected. Furthermore, nominal associations of rs182637 were found for the Color Trails Test 2 assessing executive function (*p* = 0.0212) and for Sentence Repetition/Verbal Fluency evaluating language performance (*p* = 0.0411); however, no significant interaction between rs182637 and PD was observed. While these results reached nominal significance (*p* < 0.05), they did not survive Bonferroni correction (*p* < 0.0167) and thus should be interpreted with caution. In contrast, a significant SNP effect of rs182637 on attention was detected (*p* = 0.001; effect size β coefficient = −0.046, 95% CI = −0.070 to −0.021), along with a significant SNP-diagnosis interaction (*p* = 0.011; effect size β coefficient = −0.038, 95% CI = −0.068 to −0.009), indicating that SNP rs182637 significantly influenced attention and PD moderated the association (Figure S2). Collectively, these findings suggest that rs12936340 and rs182637 contribute to individual differences in cognitive performance, with their effects potentially moderated by the presence of PD.

## Discussion

### Interpretation of findings

This cross-sectional study examined the associations between three TRPV1 SNPs, rs8065080, rs12936340, and rs182637, and cognitive performance, as well as the potential moderating role of PD. To our knowledge, this is the first study to examine the genetic polymorphisms of TRPV1 across multiple cognitive domains while simultaneously assessing the moderating effect of PD.

In the HC group, cognitive performance did not significantly differ between the TT + TC and CC genotypes of rs8065080, nor between the CC + CT and TT genotypes of rs182637. For rs12936340, no significant differences were observed across most cognitive domains between the AA + AG and GG genotypes; however, individuals with the GG genotype exhibited a trend toward better visuospatial performance (*p* = 0.032).

In contrast, the influence of TRPV1 polymorphisms was more pronounced among individuals with PD. PD patients carrying the CC genotype of rs8065080 showed a trend toward superior performance of the Naming subtest of the MoCA, reflecting better language function. Most importantly, PD patients with the GG genotype of rs12936340 showed significantly better naming performance. Moderation analyses further revealed that PD enhanced the associations between specific TRPV1 SNPs and cognitive functions. While rs8065080 showed no significant main or interactive effects, rs12936340 was significantly associated with visuospatial function and language (naming) functions, and these associations were strengthened in the presence of PD. PD also appeared to moderate the relationship between rs12936340 and executive function. Similarly, rs182637 was significantly associated with global cognitive function and attention, with PD moderating the association for attention ability. Borderline significant associations were additionally observed for executive function and for sentence repetition or verbal fluency within the language domain.

Collectively, these findings suggest that TRPV1 polymorphisms contribute to individual differences in cognitive functions, with PD exerting a moderating influence on these genetic effects. To mitigate the risk of type I errors, both nominal and Bonferroni-corrected *p*-value thresholds were reported. Findings with borderline significance should therefore be interpreted with caution and require replication in larger cohorts.

### Biological implications

TRPV1 is functionally expressed in microglia within the hippocampus and cortex, as well as in astrocytes,^24^^,^^25^ and its activity has been implicated in the regulation of inflammatory processes.^13^ Increasing evidence from animal studies suggests a robust link between TRPV1 and cognitive function. For instance, one study examining the role of TRPV1 in a mouse model of PD dementia proposed that TRPV1 influenced both spatial and reversal learning dysfunction.^26^ Another study using a mouse model of Alzheimer disease found that TRPV1 activation effectively attenuated cognitive and synaptic dysfunctions, especially improving spatial learning and memory.^15^ Moreover, TRPV1 has also been implicated in microglial autophagy, where its activation ameliorates Alzheimer disease-related learning and memory impairments.^27^

Neuroinflammation, characterized by the activation of microglia and astrocytes, plays a major role in PD pathogenesis. This activation is likely region specific and may evolve over the course of the disease, thereby contributing to both the development and progression of PD.^28^ Accumulating evidence indicates that neuroinflammation substantially contributes to cognitive disorders, including PD-associated cognitive impairment.^29^ Both human patients and rodent models of PD commonly exhibit cognitive decline, most notably affecting executive function and spatial memory.^4^^,^^26^

Our findings align with previous research, reinforcing the association between TRPV1 genetic variations and cognitive function. Specifically, we identified links to visuospatial function, attention, and language. While subtle discrepancies exist between our results and prior studies, these differences likely reflect variation in cognitive assessment sensitivity, domain focus, and the moderating effects of PD. Notably, most prior research on TRPV1 and cognition has been conducted in rodent models, where TRPV1 activation modulates synaptic plasticity and neuroinflammatory responses across brain regions, influencing cognitive domains such as spatial learning, memory, and executive function, consistent with its expression in the hippocampus and cortex. By examining TRPV1 single SNPs in a human cohort, our study provides granular, human-based evidence for TRPV1’s influence on cognition, particularly highlighting its role in visuospatial and language functions. Furthermore, the impact of PD-related neurodegeneration appears to modulate TRPV1’s influence on specific cognitive domains, such as attention and visuospatial abilities, rather than memory. Thus, our results refine and extend the current understanding of TRPV1’s contribution to cognition, underscoring the intricate interplay between TRPV1 SNPs and PD pathology, while translating rodent-based findings to human populations.

Extensive research on TRPV1 indicates that genetic variations in this receptor can result in phenotypic differences, affecting health outcomes and disease susceptibility. For example, one study identified two rare TRPV1 variants, T612M and N394del, in patients with malignant hyperthermia (MH), suggesting a potential role in MH pathogenesis.^23^ Another study reported that the TRPV1 I585V variant (rs8065080) may reduce airway sensitivity to irritants in men by contributing to airway insensitivity.^30^ This reduced sensitivity is supported by *in vitro* experiments demonstrating a reduced response to capsaicin, where higher concentrations were required to achieve an equivalent effect compared with the wild-type TRPV1.^22^ On the other hand, a Japanese study reported that the TRPV1 I585V variant significantly increased capsaicin sensitivity.^31^ These findings collectively suggest that the effects of the TRPV1 I585V variant may vary based on environmental factors or disease context, leading to different phenotypic outcomes.

Additionally, in an elderly cohort, the TRPV1 rs8065080 SNP was associated with an increased likelihood of depression, particularly in men,^32^ and another study in young adults reported that this SNP may influence salt taste sensitivity,^32^^,^^33^ indicating broader sensory implications of TRPV1 genetic variation. Although research on TRPV1 SNPs and neuroinflammation remains limited, it is plausible that these genetic variations influence neuroinflammatory mechanisms that, in turn, affect cognitive function. The present study adds to this growing body of evidence by exploring the role of TRPV1 polymorphisms in cognitive impairment associated with PD. Notably, TRPV1 SNPs exhibited significant associations with visuospatial function and language abilities, suggesting the potential impact of TRPV1 polymorphisms on cognition, with PD further moderating these effects. To our knowledge, this is the first human study to demonstrate disease-specific moderation of TRPV1-cognition associations in PD.

TRPV1 is increasingly recognized as a potential molecular target for novel PD therapies. Capsaicin, a highly selective TRPV1 agonist, has been shown to protect nigrostriatal dopamine neurons by inhibiting glial activation-mediated oxidative stress and neuroinflammation in a 1-methyl-4-phenyl-1,2,3,6-tetrahydropyridine-induced PD mouse model, thereby enhancing striatal dopaminergic function and improving behavioral recovery.^34^ Subsequent studies further elucidated TRPV1’s neuroprotective mechanisms. Specifically, TRPV1 upregulates growth differentiation factor 11, which inhibits oxidative stress, cell senescence, and apoptosis, thereby preventing dopaminergic neuronal loss in PD models.^35^ Moreover, controlled activation of TRPV1 channels on microglia using targeted photothermal agents has been shown to enhance autophagy and facilitate clearance of α-synuclein aggregates, key contributors to PD progression.^36^ In our study, TRPV1 genetic variants demonstrated significant associations with cognitive domains affected in PD, suggesting a mechanistic link between TRPV1 signaling and neurocognitive modulation. These findings extend preclinical evidence to human populations, positioning TRPV1 as a biologically plausible therapeutic target in PD. Although the present data do not yet establish direct clinical utility, they highlight TRPV1 as a promising candidate for mechanistic validation and early translational exploration. With replication in larger and longitudinal cohorts, TRPV1 genotyping may eventually inform risk stratification or tailored interventions for cognitive impairment in PD.

The present study possesses several noteworthy strengths. To our knowledge, it represents one of the most comprehensive investigations into the influence of TRPV1 SNPs on cognitive function, while also evaluating the moderating effect of PD. Furthermore, cognitive function was assessed across multiple cognitive domains, including memory, executive function, attention, language, and visuospatial abilities, ensuring a multidimensional assessment of cognitive performance. This comprehensive approach provides a more nuanced understanding of how TRPV1 genetic variations may differentially affect specific cognitive domains.

We identified significant associations between TRPV1 SNPs and specific cognitive domains, with PD potentially amplifying these genotype-phenotype relationships. Specifically, our analysis revealed that SNP rs12936340 and SNP rs182637 were significantly associated with cognitive functions, particularly visuospatial function, attention, and the naming component of language function. Moreover, the presence of PD enhanced the association between rs12936340 and naming function, and between rs182637 and attention.

In summary, we identified significant associations between TRPV1 SNPs and specific cognitive domains, with PD potentially moderating these relationships. Our analysis revealed that SNP rs12936340 and SNP rs182637 were significantly associated with cognitive functions, particularly visuospatial function, attention, and the naming subset of language function. Furthermore, PD entity enhanced the association between rs12936340 and naming function, and between rs182637 and attention, providing human-based evidence for the interaction between TRPV1 signaling and PD-related neurocognitive modulation.

### Future directions

Building on these findings, we suggest several directions for future research aimed at deepening both the mechanistic and translational understanding of TRPV1’s role in PD. First, future investigations should adopt polygenic and haplotype-based approaches, such as polygenic risk scores, to better capture the broader genetic architecture of TRPV1 and its related molecular pathways involved in neuroinflammation and neurodegeneration. These approaches may reveal cumulative genetic contributions that single-SNP analyses often fail to detect.

Second, the integration of multi-modal datasets, including genetic, neuroimaging, electrophysiological, and fluid biomarker measures, could help bridge the gap between genotype and phenotype. Such integrative frameworks would allow the identification of intermediate neural or molecular endophenotypes that mediate TRPV1’s influence on cognition in PD. Furthermore, while the current study focused on cognitive impairment as a representative non-motor symptom of PD, other neuropsychiatric manifestations, such as anxiety and mood disorders, are also important issues to be explored. Investigating the role of TRPV1 in these symptoms could broaden our understanding of its overall impact on PD. Furthermore, the potential of TRPV1 as a therapeutic target for PD-related cognitive impairments merits further exploration. Targeting TRPV1 could represent a novel approach to alleviating non-motor symptoms in PD, thereby reducing the burden on caregivers and healthcare systems.

### Limitations of the study

Despite the strengths of our study, several limitations must be acknowledged. First, due to the limited number of homozygous minor allele carriers, we employed a dominant genetic model by combining heterozygotes and homozygotes into a single group. While this approach improved statistical power and analytic stability, it limited our ability to examine genotype-specific effects in detail. Future studies with larger cohorts could enable three-group analyses that elucidate potential gene dosage influences on cognition in PD. Second, the generalizability of our findings is constrained by the recruitment of participants exclusively from southern Taiwan. Although our cohort was ethnically and regionally homogeneous, allele frequency data from public databases indicate that the minor allele frequencies of the examined SNPs (rs8065080, rs12936340, and rs182637) are broadly comparable across diverse populations. This cross-population similarity suggests that our findings are unlikely to be driven by population-specific allele distributions; nonetheless, replication in independent and ethnically diverse cohorts remains essential to confirm the generalizability of these associations. Expanding sampling beyond East Asian cohorts would help determine whether the observed genotype-phenotype relationships are population specific or reflect shared biological mechanisms. Additionally, although multiple cognitive domains were assessed, the instruments used may not have provided sufficient granularity for fine-grained cognitive profiling. Employing more comprehensive and domain-sensitive neuropsychological batteries in future research may better delineate the cognitive profiles modulated by TRPV1 in PD. Fourth, while Bonferroni correction was applied for multiple SNP comparisons, we did not apply additional corrections across the various cognitive domains, which increases the potential risk of type I error. Future studies with larger sample sizes should implement more stringent multiple-testing corrections to validate the findings. Moreover, while genetic variants are determined at birth, the cross-sectional design precludes causal inference between TRPV1 polymorphisms and cognitive decline in PD. The present findings demonstrate associations between specific TRPV1 SNPs and cognitive performance; however, longitudinal studies are needed to elucidate causal pathways linking genetic variation to cognitive trajectories. Such studies would strengthen causal inference and provide insight into how TRPV1-related mechanisms contribute to disease progression over time.

Lastly, beyond statistical associations, the functional relevance of the examined SNPs also warrants consideration. Although rs8065080 is a missense variant (I585V) that may alter TRPV1 protein conformation or activity, rs12936340 and rs182637 are intronic variants lacking direct evidence of regulatory function. Nevertheless, intronic polymorphisms within the host gene can modulate its expression by altering intragenic regulatory elements,^37^ reshaping promoter-enhancer chromatin topology,^38^ or coupling transcription with splicing.^39^ Thus, the potential regulatory roles of rs12936340 and rs182637 require further functional characterization to elucidate their contribution to TRPV1 expression and cognitive phenotypes in PD.

## Resource availability

### Lead contact

Further information and requests for resources and reagents should be directed to and will be fulfilled by the lead contact, Chun-Hsiang Tan (chtan@kmu.edu.tw), upon reasonable request.

### Materials availability

This study did not generate new unique reagents.

### Data and code availability

• Data: The data supporting the findings of this study are available within the manuscript and the supplemental information.
• Code: This study does not generate original code.
• All other items: Any additional information required is available upon reasonable request to the lead contact.
• All the associated data not provided within the paper are available on request from Chun-Hsiang Tan.

## Acknowledgments

The authors are grateful to the participants involved in this study. The study was supported by grants from the 10.13039/501100004737National Health Research Institute (NHRI-EX114-11115NC), the 10.13039/100020595National Science and Technology Council (NSTC), Taipei, Taiwan (NSTC 113-2410-H-006-093-MY2 and NSTC 113-2320-B-037 -026 -MY3), and 10.13039/100007404Kaohsiung Medical University Hospital (KMUH114-4R66).

## Author contributions

All authors fulfilled the authorship criteria, and no one who met the criteria was excluded. C.-H.T. and R.-L.Y. had the idea and designed the experiments. C.-H.T. and R.-L.Y. were involved in data collection. C.-H.T. performed data analysis. W.-S.Y. and C.-H.T. wrote the paper and took responsibility for interpreting the results. All authors critically reviewed drafts and approved the final version of this article. C.-H.T. accepted full responsibility for the work and controlled the decision to publish.

## Declaration of interests

The authors have no conflicts of interest to declare.

## STAR★Methods

### Key resources table

**Table:** 

REAGENT or RESOURCE	SOURCE	IDENTIFIER
**Biological samples**		

DNA extracted from peripheral blood leukocytes from study participants (Parkinson’s disease patients and healthy control subjects)	Collected and prepared by Dr. Chun-Hsiang Tan and Professor Rwei-Ling Yu at Kaohsiung Medical University Hospital and National Cheng Kung University Hospital	N/A

**Critical commercial assays**		

Axiom™ Genome-Wide TWB 2.0 Array Plate	Thermo Fisher Scientific / Affymetrix	GEO platform: GPL32738
BioTek Epoch 2 Microplate Spectrophotometer	Agilent Technologies	Model number: EPOCH2NS

**Deposited data**		

Raw and analyzed genotyping and cognitive data supporting findings	This paper	Available upon request from corresponding author (Dr. Chun-Hsiang Tan)

**Software and algorithms**		

IBM SPSS Statistics for Windows, v22.0	IBM Corp.	https://www.ibm.com/products/spss-statistics
PROCESS macro v4.2 for SPSS	Hayes.^40^ PROCESS Macro for SPSS, SAS, and R (Version 4.2)	https://processmacro.org/index.html
HaploView v4.2	Broad Institute (Barrett et al.^41^)	https://www.broadinstitute.org/haploview/haploview

**Other**		

Montreal Cognitive Assessment (MoCA)	Translated and validated from the original English version (Nasreddine et al.)^42^ for use in Taiwan; official Traditional Chinese version available at the MoCA website	https://www.mocatest.org
Color Trails Test (CTT)	Translated and validated from the original English version (D’Elia et al.)^43^ for use in Taiwan; distributed by Chinese Behavioral Science Corporation	https://www.mytest.com.tw/EF_CTT.aspx
Wechsler Memory Scale–Third Edition (WMS-III), Traditional Chinese Version (Logical Memory subtest)	Translated and validated from the original English version (Pearson Assessments) for use in Taiwan; distributed by Chinese Behavioral Science Corporation	https://www.mytest.com.tw/Adult_page.aspx?title=I_WMSIII

### Experimental model and study participant details

#### Study participants and design

This cross-sectional study enrolled 401 individuals of East Asian ethnicity. Participants were recruited from Kaohsiung Medical University Hospital (approval number: KMUHIRB-G(II)-20160001), National Cheng Kung University Hospital (approval number: --/B-ER-104-082), and the surrounding community between 2017 and 2023. The study population comprised 274 HC and 127 individuals with PD. Each participant underwent a single standardized assessment.

#### Inclusion and exclusion criteria

PD diagnoses were established by neurologists based on the Movement Disorder Society diagnostic criteria for PD,^44^ excluding those with atypical parkinsonism. community-dwelling volunteers aged older than 50 years. Exclusion criteria for both groups included a history of brain surgery, comorbid psychiatric or neurological disorders, or inability to provide informed consent, all of which were verified by neurologists.

#### Reporting of sex and ethical oversight

The sex distribution between PD and HC groups was not significantly different, confirming homogeneity across groups (see Table 1). Furthermore, the influence of sex on the primary cognitive outcomes was rigorously evaluated by including sex (along with age and education) as a covariate in all primary statistical models. In these models, sex did not exhibit a significant main effect on any of the cognitive performance outcomes. Furthermore, all participants were of East Asian ethnicity, providing ethnic homogeneity for this genetic study. All study procedures were approved by the Institutional Review Boards of Kaohsiung Medical University Hospital and National Cheng Kung University Hospital. All participants provided written informed consent prior to their inclusion.

#### Ethics approval and consent to participate

All study procedures were approved by the ethical research committee of Kaohsiung Medical University Hospital (approval number: KMUHIRB-G(II)-20160001), and National Cheng Kung University Hospital (approval number: --/B-ER-104-082), and all methods were performed following the approved guidelines. The participants’ written informed consent was obtained before enrollment, following the ethical standards outlined in the 1975 Declaration of Helsinki.

### Method details

#### Cognitive assessment

Global cognitive function was evaluated using the Montreal Cognitive Assessment (MoCA),^45^ complemented by additional tests to evaluate five cognitive domains: executive function, visuospatial function, memory, attention, and language.^46^ Executive function was evaluated using the Color Trail Tests 1 and 2. The visuospatial function was assessed through MoCA subtests, including Alternating Trail Making, Cube, and Clock. Immediate and delayed memory were evaluated using the Logical Memory subtest from the Wechsler Memory Scale-Third Edition.^47^ Attention was assessed using the attention subtest of MoCA, and language ability was evaluated with the MoCA Naming, Sentence Repetition, and Verbal Fluency subtests. Consistent with the domain structure validated by MoCA and supported by subsequent psychometric work, the Naming, Sentence Repetition, and Verbal Fluency subtests were treated as complementary indicators of the language domain, rather than as independent constructs. All participants underwent cognitive testing in a quiet room under standardized conditions to minimize distractions. Trained examiners administered the tests in a fixed order to ensure consistency, following established scoring criteria. Sufficient time was provided for all tasks. MoCA is scored from 0 to 30, with higher scores indicating better cognitive performance. The Color Trail Tests assess processing speed, where longer completion times suggest greater impairment. The Logical Memory subtest of the Wechsler Memory Scale-Third Edition measures recall ability, with higher scores reflecting better memory performance.

#### Genetic testing

Peripheral blood leukocytes were collected from all participants to extract genomic DNA. DNA concentration and purity were measured using a BioTek Epoch 2 Microplate Spectrophotometer, with an A260/A280 ratio maintained at approximately 1.8. TRPV1 SNPs genotyping was performed using the Affymetrix GeneChip platform (C2-58 Axiom Genome-Wide TWB 2.0 Array Plate), with quality control ensuring call rates above 98%. The Hardy-Weinberg equilibrium test was used to detect genotyping or sampling errors in all SNPs. Minor allele frequency was calculated, and only SNPs with a minor allele frequency higher than 10% were included for further analysis (Table 1). Using the genotyping results, we constructed the pairwise LD pattern among the TRPV1 SNPs, including rs8065080, rs12936340, rs3744684, rs79821076, and rs182637, with HaploView 4.2 software and employed the solid spine of LD method to determine the LD block.

### Quantification and statistical analysis

#### Statistical software and data processing

Statistical analyses were conducted using the IBM Statistical Package for the Social Sciences Statistics for Windows (Version 22.0). The Kolmogorov–Smirnov test was used to assess the normality of variables. Quantitative variables were expressed as means ± standard deviation (SD), and qualitative variables were expressed as percentages.

#### Statistical tests

T-tests were applied to normally distributed variables, while Mann–Whitney U-tests were used to measure non-normally distributed variables. Chi-square tests were applied for categorical variables such as sex ratio.

#### Genetic modeling and exclusion criteria

To address insufficient statistical power stemming from small sample sizes, we employed a dominant genetic model. This involved combining participants carrying the minor allele (both homozygotes and heterozygotes) into a single group for analysis, which was then compared with participants homozygous for the major allele, to ensure analytic stability. Participants lacking genotype data for rs182637, rs3744684, or rs79821076 were excluded only from the analyses involving the respective SNP, but were retained for analyses of other SNPs.

#### Moderation analysis

The primary analysis examining the interplay between genetics and diagnosis was performed using the PROCESS macro (version 4.2) for SPSS. This was used to examine the impact of TRPV1 SNPs on cognitive function and to evaluate whether the genetic effects differed between the PD and HC groups. Disease status was set as the moderator variable (W), cognitive assessment results were the dependent variables (Y), and TRPV1 SNPs were the independent variables (X).

#### Covariates and correction

Age, sex, and years of education were included as covariates in all primary analyses to control for potential confounding effects. To account for multiple comparisons across the three primary SNPs examined, a Bonferroni correction was applied, setting the statistical significance threshold at p < 0.0167 (derived from p = 0.05/3). Findings with p values between the nominal threshold (p < 0.05) and the Bonferroni-corrected threshold (p < 0.0167) were interpreted as suggestive or borderline significant.
