# Supplementary material for: Association of TRPV1 genetic variants with cognitive functions in Parkinson disease
Source: iScience. 2025 Dec 24;29(1):114538. doi: 10.1016/j.isci.2025.114538 (PMC12818065; doi:10.1016/j.isci.2025.114538)
Supplement: Document S1. Figures S1–S2 and Tables S1–S3 [file mmc1.pdf]

iScience, Volume *29*

Supplemental information

Association of TRPV1 genetic variants with cognitive functions in Parkinson disease

Wei-Shan Yao, Rwei-Ling Yu, and Chun-Hsiang Tan

Figure S1: Illustration of the moderating effect of PD diagnosis on the association between rs12936340 genotype and MoCA Naming scores

Moderating effect of Parkinson's disease on the association between rs12936340 and

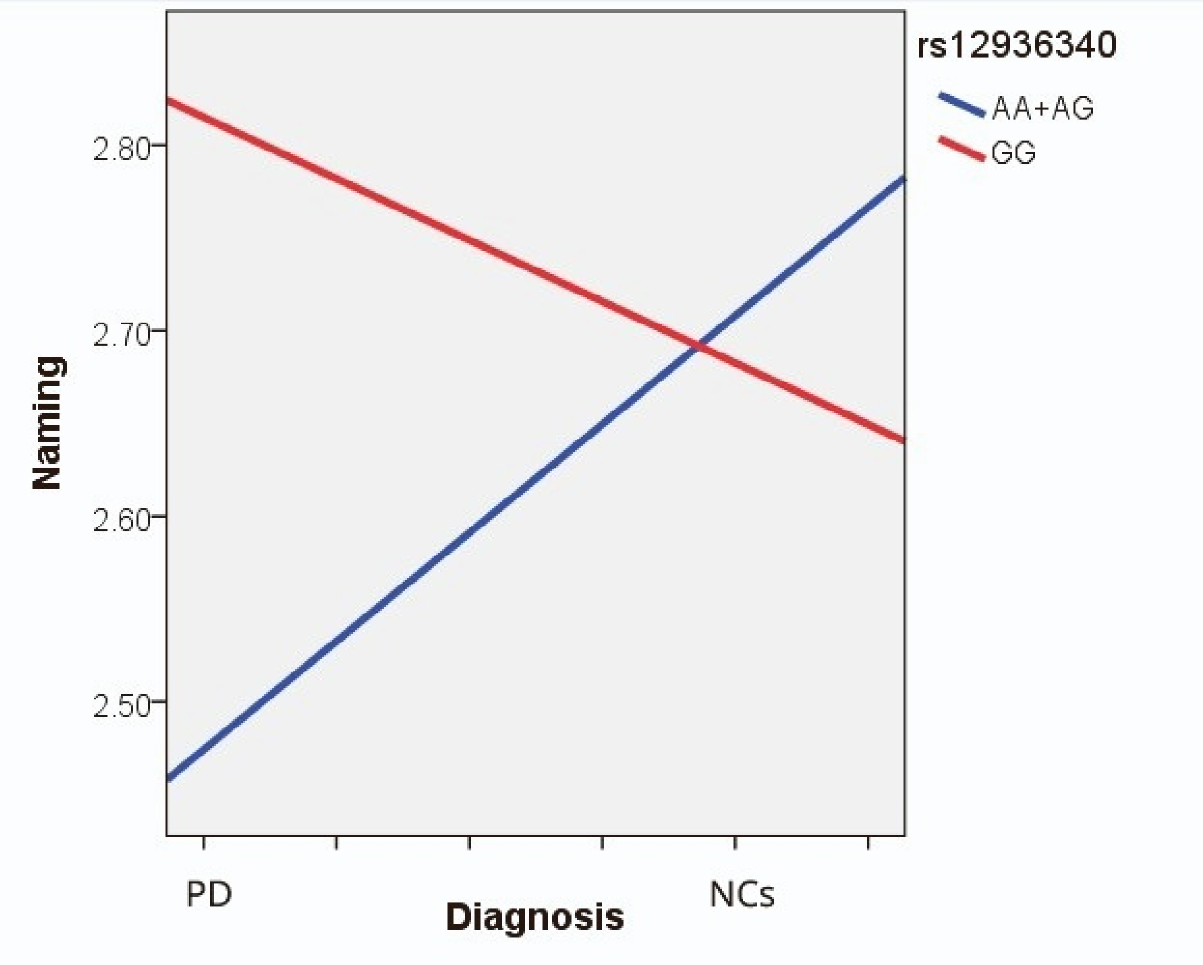

MoCA naming scores. Interaction between rs12936340 genotypes and Parkinson's disease (PD) status on the naming subtest of the Montreal Cognitive Assessment (MoCA). The effect of rs12936340 genotypes on naming performance was significantly moderated by PD diagnosis. Among healthy controls (NCs), individuals carrying the A allele (AA + AG) and those with the GG genotype showed comparable naming performance. In contrast, among individuals with PD, A-allele carriers exhibited significantly poorer naming scores than those with the GG genotype. This pattern indicates that the presence of PD amplifies the deleterious association between the rs12936340 A allele and language performance. Moderation Analysis: Interaction $\beta = -0.047$, 95% CI: -0.070-0.021, $p < 0.001$. Main effect of genotype: $\beta = 0.216$, 95% CI: 0.095-0.337, $p < 0.001$. N=401 participants.

Figure S2: The moderating effect of PD diagnosis on the association between rs182637 genotype and MoCA Attention scores

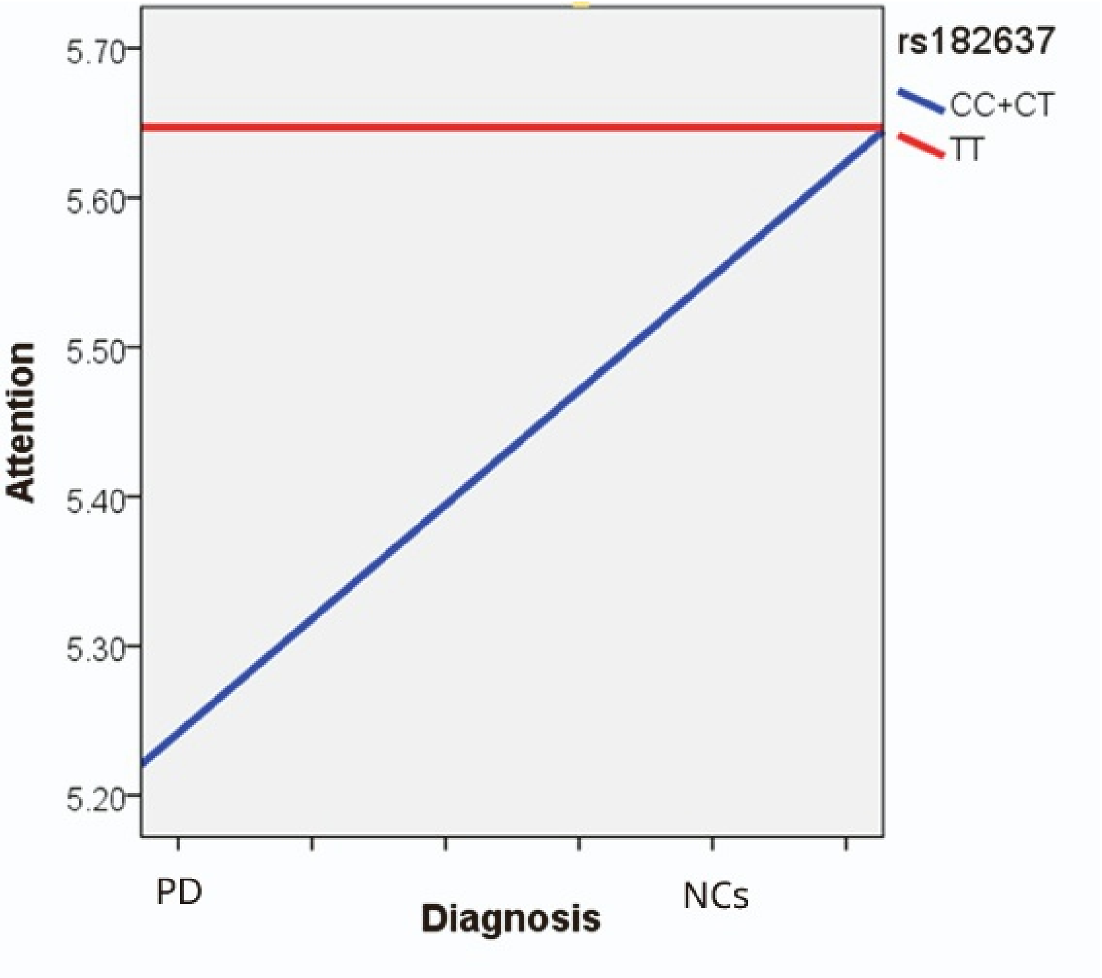

Moderating effect of Parkinson's disease on the association between rs182637 and MoCA attention scores. Interaction between rs182637 genotypes and Parkinson's disease (PD) status on the attention subtest of the Montreal Cognitive Assessment (MoCA). The influence of rs182637 on attention performance was significantly moderated by PD diagnosis. Among healthy controls (NCs), individuals carrying the C allele (CC + CT) and those with the TT genotype showed comparable attention scores. In contrast, among individuals with PD, carriers of the C allele demonstrated lower attention scores than those with the TT genotype, indicating that PD amplifies the detrimental effect of the rs182637 C allele on attentional performance. Moderation Analysis: Interaction β = -0.038, 95% CI: -0.068~-0.009, p = 0.011. Main effect of genotype: β = -0.046, 95% CI: -0.070~-0.021, p = 0.001. N=401 participants

Table S1. Effects of TRPV1 SNPs in Linkage Disequilibrium with rs12936340 on the cognitive functions of participants in the healthy control group

SNP	rs3744684 (n=273)	rs3744684 (n=273)	rs3744684 (n=273)	rs79821076 (n=274)	rs79821076 (n=274)	rs79821076 (n=274)
Genotype	GG+GA (n=90)	AA (n=183)	Statistic	AA+AG (n=85)	GG (n=189)	Statistic
Age (years)	66.02±6.470	64.57±7.010	p=0.200	64.68±7.160	65.24±6.723	p=0.611
Male/Female	24/66	49/134	p=0.985	27/58	47/142	p=0.235
Education (years)	11.82±3.686	12.56±3.659	p=0.080	12.56±4.055	12.22±3.501	p=0.209 r= -0.076
Global cognitive screening: Montreal Cognitive Assessment	25.06±3.413	25±3.822	p=0.791	25.16±3.631	24.93±3.719	p=0.587
Executive function: Color Trails Test 1	55.21±23.120	54.69±28.355	p=0.458	53.6±26.254	55.28±26.957	p=0.702
Executive function: Color Trails Test 2	119.41±49.089	113.49±54.857	p=0.118	110.42±46.935	117.57±55.390	p=0.384
Visuospatial function / Executive function: Alternating Trail Making / Cube / Clock (Montreal Cognitive Assessment)	4.2±0.902	4.35±0.913	p=0.101	4.26±0.990	4.31±0.877	p=0.919
Memory: Wechsler Memory Scale-III Immediate Logical Memory	34.34±12.062	33.53±11.205	p=0.906	34.73±11.660	33.39±11.372	p=0.582
Memory: Wechsler Memory Scale-III Delayed Logical Memory	20.86±9.282	20.82±8.989	p=0.690	21.72±9.201	20.35±9.043	p=0.388
Attention: Attention (Montreal Cognitive Assessment)	5.64±0.641	5.64±0.696	p=0.832	5.62±0.636	5.65±0.697	p=0.458
Language: Naming (Montreal Cognitive Assessment)	2.69±0.630	2.66±0.650	p=0.695	2.74±0.515	2.63±0.691	p=0.381
Language: Sentence Repetition / Verbal Fluency (Montreal Cognitive Assessment)	2.41±0.777	2.35±0.790	p=0.488	2.46±0.749	2.33±0.798	p=0.178

$* = p < 0.0167$

Table S2. Effects of TRPV1 SNPs in Linkage Disequilibrium with rs12936340 on the cognitive functions of participants with Parkinson's disease

SNP	rs3744684 (n=125)	rs3744684 (n=125)	rs3744684 (n=125)	rs79821076 (n=126)	rs79821076 (n=126)	rs79821076 (n=126)
Genotype	GG+GA (n=42)	AA (n=83)	Statistic	AA+AG (n=46)	GG (n=80)	Statistic
Age (years)	68.31±7.649	65.27±7.754	p=0.023	66.89±8.486	66.01±7.426	p=0.486
Male/Female	27/15	56/27	p=0.723	35/11	49/31	p=0.090
Education (years)	12.76±3.968	12.16±4.059	p=0.572	12.33±3.853	12.43±4.136	p=0.969
Global cognitive screening: Montreal Cognitive Assessment	23±4.102	23.06±3.980	p= 0.958	23.39±4.118	22.81±3.930	p=0.390
Executive function: Color Trails Test 1	79.83±44.165	69.72±44.374	p=0.109	72.79±39.699	73.64±46.939	p=0.859
Executive function: Color Trails Test 2	158.08±88.218	143.93±95.050	p=0.299	152.48±71.981	149.97±107.242	p=0.318
Visuospatial function / Executive function: Alternating Trail Making / Cube / Clock (Montreal Cognitive Assessment)	3.69±1.179	4.1±1.019	p=0.056	4±1.135	3.95±1.066	p=0.642
Memory: Wechsler Memory Scale-III Immediate Logical Memory	29.64±12.131	29.08±12.413	p=0.603	27.93±12.643	30±11.997	p=0.269
Memory: Wechsler Memory Scale-III Delayed Logical Memory	16.88±9.253	16.48±9.891	p=0.800	15.76±10.550	17.06±9.066	p=0.439
Attention: Attention(Montreal Cognitive Assessment)	5.36±0.983	5.41±1.000	p=0.722	5.54±0.751	5.31±1.098	p=0.306
Language: Naming (Montreal Cognitive Assessment)	2.62±0.731	2.76±0.484	p=0.458	2.8±0.500	2.66±0.615	p=0.130
Language: Sentence Repetition / Verbal Fluency (Montreal Cognitive Assessment)	2.14±0.899	2.05±0.896	p=0.549	2.09±0.915	2.08±0.883	p=0.919

$* = p < 0.0167$

Table S3. Moderation between TRPV1 SNPs in Linkage Disequilibrium with rs12936340 and disease entity on cognitive functions

	rs3744684 SNP effect	rs3744684 SNP-diagnosis interaction	rs79821076 SNP effect	rs79821076 SNP-diagnosis interaction
Global cognitive screening:<br>Montreal Cognitive Assessment	p= 0.850	p= 0.381<br>1-β=0.066	p= 0.125	p= 0.234<br>1-β=0.115
Executive function:<br>Color Trails Test 1	p= 0.178	p= 0.100<br>1-β=0.231	p= 0.661	p= 0.678<br>1-β=0.027
Executive function:<br>Color Trails Test 2	p= 0.551	p= 0.476<br>1-β=0.049	p= 0.994	p= 0.862<br>1-β=0.020
Visuospatial function / Executive function:<br>Alternating Trail Making / Cube / Clock (Montreal Cognitive Assessment)	**p= 0.009***<br>***β*= 0.252**<br>**CI: 0.062~0.440**	p= 0.044<br>1-β=0.362	p= 0.501	p= 0.322<br>1-β=0.081
Memory:<br>Wechsler Memory Scale-III<br>Immediate Logical Memory	p= 0.953	p= 0.438<br>1-β=0.055	p= 0.430	p= 0.337<br>1-β=0.079
Memory:<br>Wechsler Memory Scale-III<br>Delayed Logical Memory	p= 0.839	p= 0.705<br>1-β=0.026	p= 0.630	p= 0.381<br>1-β=0.066
Attention:<br>Attention (Montreal Cognitive Assessment)	p= 0.542	p= 0.326<br>1-β=0.081	p= 0.037	p= 0.045<br>1-β=0.355
Language:<br>Naming (Montreal Cognitive Assessment)	p= 0.179	p= 0.110<br>1-β=0.218	p= 0.318	p= 0.701<br>1-β=0.025
Language:<br>Sentence Repetition / Verbal Fluency (Montreal Cognitive Assessment)	p= 0.563	p= 0.796<br>1-β=0.019	p= 0.678	p= 0.841<br>1-β=0.019

$* = p < 0.0167$; β = standardized regression coefficient; $1 - \beta$ = statistical power (where β represents the probability of a Type II error); CI = 95% confidence interval.
